# *Chlamydia pneumoniae* Antibodies and C-Reactive Protein Levels in Patients with Abdominal Aortic Aneurysms

**DOI:** 10.1155/2013/212450

**Published:** 2013-12-28

**Authors:** M. A. Sharif, D. A. McDowell, S. A. Badger

**Affiliations:** ^1^Department of Vascular and Endovascular Surgery, Belfast City Hospital, Belfast BT9 7AB, UK; ^2^School of Health Sciences, University of Ulster, Shore Road, Newtownabbey BT37 0QB, UK

## Abstract

*Introduction*. The study aim was to assess the relationship between the presence of antibodies to *Chlamydia pneumoniae* and abdominal aortic aneurysm (AAA) incidence. *Patients and Methods*. Consecutive AAA patients and AAA-free controls were recruited prospectively. Serum samples from both groups were examined to determine Immunoglobulin (Ig) A and IgG titres against *Chlamydia pneumoniae* by ELISA and C-reactive protein (CRP) concentrations. Results were expressed as mean (SD) or median (IQR) and compared using *χ* ^2^ and Mann-Whitney *U* tests. A *P* value of <0.05 was considered statistically significant. *Results*. Each study group (AAA/nAAA) comprised 250 patients. 196 (78.7%) AAA patients had positive IgA antichlamydial antibody titres, compared to 181 (72.4%) in the control group (*P* = 0.008, OR 2.0, 95% CI 1.2–3.5). However, positive IgG antibody titres were similar (191 versus 203; *P* = 0.222, OR 0.7, 95% CI 0.4–1.3). Average CRP concentrations were higher in AAA individuals. IgA or IgG antibody titres were not related to CRP concentrations. *Conclusions*. These results demonstrated that the frequent incidence of *Chlamydia pneumoniae* antibodies within the general population makes it difficult to relate its presence to AAA development, despite the high IgA antibody titres. In addition, raised CRP concentrations in AAA patients are not related to the presence of antichlamydial antibodies.

## 1. Introduction

The aetiology of abdominal aortic aneurysms (AAAs) is multifactorial. However, enzymatic degradation of matrix proteins of the aortic wall by matrix metalloproteinases (MMPs) may play an important role in aneurysmal formation. Thus, inhibition of these MMPs reduces aortic wall degeneration and retards the growth of aneurysms [1]. Indomethacin, a nonspecific cyclooxygenase inhibitor, may attenuate aortic aneurysm growth by decreasing prostaglandin E2 and MMP expression via the COX2 isoform of cyclooxygenase [2]. Similarly, batimastat, a specific inhibitor of MMPs, may reduce elastin degradation in the aortas of rats given elastase, with reduced aneurysm growth and a reduction in the inflammatory response in the aortic wall [3]. In a case-control study, aneurysms expanded more slowly in patients treated with nonsteroidal anti-inflammatory drugs than in untreated patients [4].

Infection with *Chlamydia pneumoniae* has been suggested to contribute to MMP production and aneurysm expansion. In a double-blind randomised study, the eradication of chlamydia by the administration of doxycycline was found to reduce serum concentrations of C-reactive protein and aneurysm growth [5]. In a phase two study prolonged administration of doxycycline was shown to be safe and well tolerated by patients with small asymptomatic AAAs and was associated with a gradual reduction in plasma MMP-9 levels [6]. These observations suggest that doxycycline eradicates *Chlamydia pneumoniae* infection, reducing the rate of growth of aneurysms.

The aim of this prospective case-control study was to assess the relationship between *Chlamydia pneumoniae* antibodies and the incidence of AAA in Northern Ireland and to determine the relationship, if any, between raised C-reactive protein levels and AAA in people with antichlamydial antibodies.

## 2. Patients and Methods

### 2.1. Patient Recruitment and Inclusion Criteria

Ethical approval was obtained from the Northern Ireland Research Ethics Committee and Belfast City Hospital Trust provided the clinical indemnity for this case control, prospective, single centre study. The control (nAAA) group was recruited from those participants of the Northern Ireland AAA screening trial who had normal aortic diameters, that is, a longitudinal scan anterior-posterior diameter of less than 3.0 cm, as determined using the Sonosite 180 Plus system (Sonosite Inc., Bothwell, WA, USA). The patients in the AAA group were recruited from those attending the vascular outpatients clinic for routine follow-up of small aneurysms and those admitted in hospital for surgical treatment of large AAA. Aneurysms were classed as small (3.0–4.4 cm), medium (4.5–5.5 cm), or large (>5.5 cm) according to the maximum diameter. Informed consent was obtained in writing from all participants.

Separate blood samples were collected for enzyme-linked immunosorbent assay (ELISA) and C-reactive protein assay (CRP) in clot activator tubes (Vacutainer, MD367954, MidMeds Limited, Unit 71, Hillgrove Business Park, UK).

A medical questionnaire was completed for all the recruited participants, including a detailed assessment of cardiovascular risk factors such as hypertension, diabetes mellitus, hypercholesterolemia, smoking status, coronary artery disease (CAD, defined as evidence of previous myocardial infarction, coronary artery bypass grafting, percutaneous coronary intervention, or medical treatment for angina), cerebrovascular accident (CVA, defined as evidence of either a previous stroke or transient ischemic attack), and peripheral arterial disease (PAD, defined as an ankle brachial pressure index (ABPI) of <0.80 with or without symptoms of intermittent claudication). Other elements of the questionnaire included previous history of inflammatory disease, chronic obstructive airway disease (COAD), and family history of AAA. The questionnaire also included smoking history, differentiating current, former, and nonsmokers as outlined in the MONICA project by the World Health Organisation [7].

### 2.2. Laboratory Analyses

#### 2.2.1. ELISA Estimation of IgA and IgG Antibodies to *Chlamydia pneumoniae*

Recovered blood samples were immediately centrifuged at 2000 rpm for 10 minutes at 4°C. The resultant serum was dispensed into sterile endotoxin-free tubes (Nunc 363401, Intermed, Rosklide, Denmark) and stored at −80°C. Strict aseptic precautions were observed during sample recovery, preparation, and assay.

The concentrations of IgA and IgG antibodies to *Chlamydia pneumoniae* were measured using ELISA kits (Ani Labsystems Ltd, Vantaa, Finland), following the manufacturer's instructions. All samples were analysed in duplicate and a mean of these values was recorded as the representative value. Absorbance was measured at 450 nm and the concentrations of enzyme immunounit (EIU) of IgA and IgG were recorded as negative (EIU <8), borderline (EIU 8–12), or positive (EIU >12) values.

#### 2.2.2. C-Reactive Protein Assay

The C-reactive protein concentrations were measured using the Tina-quant CRP latex particle-enhanced immunoturbidimetric assay (Roche Diagnostics Limited, Burgess Hill, West Sussex, United Kingdom).

### 2.3. Statistical Analysis

The data were analysed using SPSS 15.0.1 (SPSS Inc, Chicago, IL) for Windows (Microsoft Inc, Redmond, WA). Age and BMI were expressed as mean and standard deviation. Vascular risk factors, comorbid conditions, and immunoglobulin titres were expressed as numbers (percentage) and chi-square test was used to compare the difference between AAA and nAAA groups. Mann-Whitney *U* test was used to compare CRP levels between the two groups and to compare the CRP levels with the immunoglobulin titres within groups. Spearman's rank correlation coefficient was used to correlate age with antibody titres and CRP. A *P* value of <0.05 was considered statistically significant.

## 3. Results

A total of 500 patients were recruited with 250 in each group during two years of the study period. The serum sample of one patient from the AAA group was missing and hence it was excluded from ELISA and CRP analyses.

### 3.1. Demographic Details and Risk Factors

Patients in the AAA and control groups had similar preoperative risk factors, except for age (*P* = 0.0001), current smoking status (*P* = 0.0002), history of CAD (*P* = 0.001), inflammatory disease (*P* = 0.012), and family history of AAA (*P* = 0.026). The AAA group was older and had an increased incidence of comorbid risk factors (Tables 1 and 2). The only significant correlation between these risk factors and the study end points, namely, IgA, IgG, and CRP, was observed between age and IgG levels in the control group (*r*_*s*_ = 0.145, *P* = 0.022). However, this correlation was not significant in the AAA group (*r*_*s*_ = 0.044, *P* = 0.488).

### 3.2. IgA and IgG Antichlamydial Antibody Titres in Control and AAA Groups

Borderline and positive IgA titres were observed in both patient groups. However, those in the AAA group had higher incidences of both the borderline and positive IgA titres, *P* = 0.022 and 0.0008, respectively (Table 3). Similarly, borderline and positive IgG titres were observed in both groups, but no significant difference was seen between the control and the AAA groups, *P* = 0.743 and 0.223, respectively (Table 4).

### 3.3. IgA Antichlamydial Antibody Titres and the Size of the AAA

**Figure 1:**
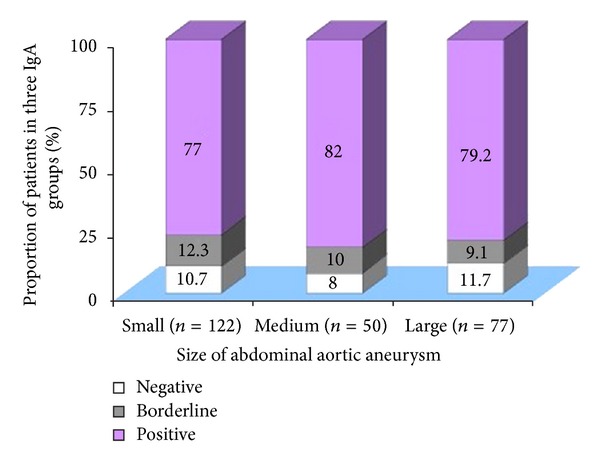
Comparison of the size of AAA based on IgA antichlamydial antibody titres. *χ* ^2^ = 1.007, df = 4, and *P* = 0.909. *n*: numbers in each group.

Analysis of IgA titres in 249 patients with AAA demonstrated that there was no significant difference in the distribution of IgA titres amongst aneurysms of small, medium, or large size (*χ* ^2^ = 1.007, df = 4, and *P* = 0.909; see Figure 1).

### 3.4. C-Reactive Proteins and AAA

**Figure 2:**
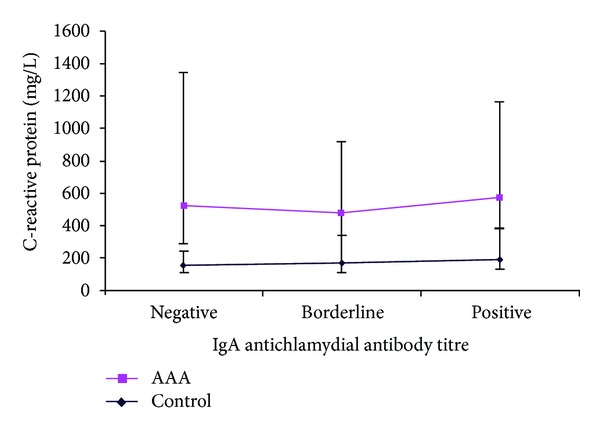
C-reactive protein in control versus AAA groups at different IgA antichlamydial antibody titres: negative (*P* = 0.002), borderline (*P* = 0.018), and positive (*P* = 0.0001) expressed as median and IQR (Mann-Whitney *U* test).

C-reactive protein levels were significantly higher in the AAA group as compared to the nAAA group as follows: for negative IgA titres: 367 mg/L versus 152 mg/L (*P* = 0.002), borderline IgA titres: 310 mg/L versus 170 mg/L (*P* = 0.018), and positive IgA titres: 390 mg/L versus 192 mg/L (*P* = 0.0001; see Figure 2).

**Figure 3:**
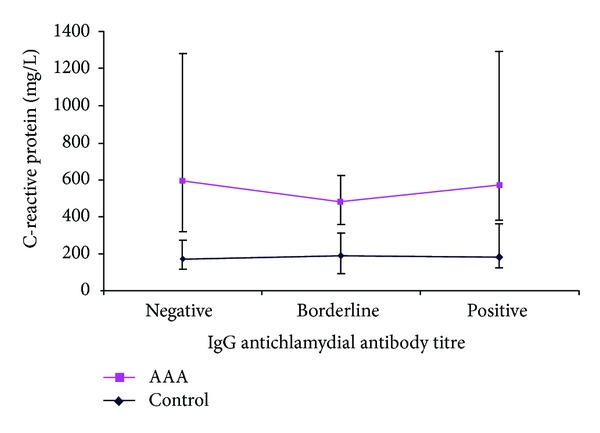
C-reactive protein in control versus AAA groups at different IgG anti chlamydial antibody titres: negative (*P* = 0.003), borderline (*P* = 0.055), and positive (*P* = 0.0001) expressed as median and IQR (Mann-Whitney *U* test).

C-reactive protein levels were also elevated in the AAA group as compared to the control group in patients with either negative or positive IgG antibody titres as follows: for negative IgG titres: 421 mg/L versus 172 mg/L (*P* = 0.003), and positive IgG titres: 390 mg/L versus 183 mg/L (*P* = 0.0001). However, for the borderline IgG titres, there was no significant difference: 294 mg/L versus 188 mg/L (*P* = 0.055; see Figure 3).

### 3.5. C-Reactive Proteins and Antibody Titres

In the control group, C-reactive protein levels were significantly higher in patients with positive IgA titres than in patients with negative IgA titres (*P* = 0.012). However, in this group, there was no significant difference in CRP levels between the borderline and negative IgA titres. Similarly, in the AAA group, no significant difference in CRP was observed with raised IgA titres. In addition, there was no significant difference in CRP levels with raised IgG titres in both the control and AAA groups (Table 5).

## 4. Discussion

*Chlamydia pneumoniae* is an obligate intracellular prokaryotic pathogen, which can infect and survive in a wide variety of host cell types, including vascular endothelium, arterial smooth muscle cells, and circulating blood cells [8]. Exposure to the organism is very common, frequently leading to a chronic state of persistent infection. Approximately half of individuals test seropositive at the age of 20 years, rising to 80% and 70% of men and women, respectively, at the age of 65 years [9].

The association of *Chlamydia pneumoniae* with atherosclerosis was first suggested in 1996 following analysis of data from heart transplant studies [10]. In 1997, Juvonen et al. discovered that the aortic wall tissue of aneurysms was frequently infected by *Chlamydia pneumoniae,* suggesting a “potential aetiological role” [11]. Subsequently, Petersen et al. showed in a larger case control study that infrarenal AAAs had a significantly higher incidence of *Chlamydia pneumoniae* in the aortic tissue compared to deceased people with normal aortic diameter [12]. Several other immunohistochemical studies produced further evidence of direct infection of aortic wall tissue, but all were rather underpowered [13, 14]. However, despite the accumulation of a large amount of evidence, it remains extremely difficult to be certain as to the role of *Chlamydia pneumoniae* in the initiation or acceleration of atherosclerosis. Such uncertainties relate to the high background prevalence of infection and the multifactorial and chronic nature of atherosclerosis. While the known prevalence of *Chlamydia* in females is higher, the small number of females included in this study reflects the gender difference in aneurysmal disease prevalence, with approximately fivefold increase in males. However, the current results may be different if more female patients were recruited for a future study.

A Polish study involving 52 AAA patients and 30 controls detected serological markers of *Chlamydia pneumoniae* in 86.5% of AAA patients and 33.3% in the controls [15]. This is an interesting contrast to the present study, where 72.4% and 81.2% proved positive for IgA and IgG, respectively, in the control group. It is, therefore, evident that the Northern Ireland population has a particularly high level of background chronic exposure to *Chlamydia pneumoniae*. Such high background levels make it more difficult to establish a clear causal relationship. Although statistical significance was reached in IgA comparisons, the actual percentage difference was small. Inclusion of the IgG results, which demonstrated more seropositivity in the control group, makes the association more tenuous.

Elevation of inflammatory markers in AAA patients is widely recognised. Our group has previously reported such effects in the patients of the present study and also noted that higher CRP concentrations were related to larger AAAs but not influenced by genetic differences [16]. Such elevation has now been demonstrated to be independent of the serum concentrations of *Chlamydia pneumoniae* antibodies, indicating that the chronic state of infection does not invoke any IgA or IgG related inflammatory response, which might explain the state of chronic aortic inflammation associated with aneurysmal disease. Toll-like receptor 4 (TLR4) recognises *Chlamydia pneumoniae* and results in the proliferation of vascular smooth muscle cells. This TLR4 activation and associated upregulation in atherosclerotic plaques give an insight into the aneurysmal development and vascular remodelling, although the evidence is only weak at present, requiring further research [17, 18].

Debate persists as to whether the *Chlamydia pneumoniae* infection is associated with initiation or progression of the disease. Lindholt et al. studied 139 men with small AAAs [19]. They observed that in men with higher IgA titres, AAA growth was 48% faster than in men with lower titres, over a mean follow-up period of 2.7 years. However, that study did not include a control group to establish the background exposure to *Chlamydia pneumoniae* in that locality. While the current study did not assess growth rate, there was no evidence of an association between immunoglobulin concentration and AAA maximal diameter. This is an important finding, especially as the current study included significantly larger case and control cohort sizes than those of almost all previous studies.

In conclusion, these results demonstrate that *Chlamydia pneumoniae* infection is very common in Northern Ireland. Such higher levels of infection make it difficult to establish the role of these infections in the initiation and/or development of AAA, despite the statistically greater incidence of detectable IgA antibody titres. In addition, the study has established that raised C-reactive protein levels in AAA patients are not related to either IgA or IgG antichlamydial antibodies.

## Figures and Tables

**Table 1:** Patient characteristics in the control and AAA groups.

	Control group (*n* = 250)	AAA group (*n* = 250)	*P* value
Gender (M : F)	238 : 12	229 : 21	0.105
Age (years)	68.7 (5.0)	71.9 (6.8)	0.0001
BMI	26.2 (3.9)	25.9 (3.4)	0.54

Vascular risk factors (%)			
Hypertension	49.2	47.6	0.72
DM	14.3	10.1	0.16
High cholesterol	32.2	35.1	0.50
Current smoking	9.8	21.9	0.0002
Ex-smoking	46.1	48.6	0.59
CAD	33.5	47.8	0.001
MI	20.8	27.9	0.07
CVA	7.8	6.5	0.58
PAD	26.5	27.9	0.73

Other comorbidities (%)			
FH AAA	11.5	18.7	0.03
Inflammatory diseases	9.4	17.1	0.01
COAD	4.5	8.6	0.07

AAA: abdominal aortic aneurysm; BMI: body mass index; DM: diabetes mellitus; CAD: coronary artery disease; MI: myocardial infarction; CVA: cerebrovascular accident; PAD: peripheral arterial disease; COAD: chronic obstructive airway disease.

**Table 2:** Inflammatory diseases in the control and AAA groups.

Condition	Control group (*n* = 23)	AAA group (*n* = 42)
Osteoarthritis	14	11
Rheumatoid arthritis	0	3
Gout	3	4
IBD	2	3
SLE	0	1
Inflammatory AAA	0	1
Others	4	19

**Table 3:** Comparison of control and AAA groups based on IgA antichlamydial antibody titres. *χ* ^2^ = 7.89, df = 2, and *P* = 0.019. The numbers in parenthesis represent percentage.

IgA titre	Control group (*n* = 250)	AAA group (*n* = 249)	OR	95% CI	*P* value
EIU <8 (negative)	48 (19.2)	26 (10.4)	1.00	—	—
EIU 8–12 (borderline)	21 (8.4)	27 (10.9)	2.4	1.1–5.4	0.022
EIU >12 (positive)	181 (72.4)	196 (78.7)	2.0	1.2–3.5	0.008

AAA: abdominal aortic aneurysm; IgA: immunoglobulin A; *χ* ^2^: chi-square; df: degree of freedom; OR: odds ratio; CI: confidence interval; *n*: number; EIU: enzyme immunounit.

**Table 4:** Comparison of control and AAA groups based on IgG antichlamydial antibody titres. *χ* ^2^ = 1.62, df = 2, and *P* = 0.444. The numbers in parenthesis represent percentage.

IgG titre	Control group (*n* = 250)	AAA group (*n* = 249)	OR	95% CI	*P* value
EIU <30 (negative)	31 (12.4)	40 (16.1)	1.00	—	—
EIU 30–45 (borderline)	16 (6.4)	18 (7.2)	0.9	0.4–2.2	0.743
EIU >45 (positive)	203 (81.2)	191 (76.7)	0.7	0.4–1.3	0.223

AAA: abdominal aortic aneurysm; IgG: Immunoglobulin G; *χ* ^2^: chi-square; df: degree of freedom; OR: odds ratio; CI: confidence interval; *n*: number; EIU: enzyme immunounit.

**Table 5:** Comparison of C-reactive protein levels with IgA and IgG antichlamydial antibody titres within the control and the AAA groups. The numbers in the table represent *P* values (Mann-Whitney *U* test).

	Control	AAA
Negative versus borderline IgA	0.840	0.669
Negative versus positive IgA	0.012*	0.776
Negative versus borderline IgG	0.840	0.614
Negative versus positive IgG	0.355	0.667

IgA: immunoglobulin A; IgG: immunoglobulin G; AAA: abdominal aortic aneurysm.

*Significant result.
